# Symmetry Breaking as a Basis for Characterization of Dielectric Materials

**DOI:** 10.3390/s25020532

**Published:** 2025-01-17

**Authors:** Dubravko Tomić, Zvonimir Šipuš

**Affiliations:** University of Zagreb Faculty of Electrical Engineering and Computing, Unska 3, 10000 Zagreb, Croatia; zvonimir.sipus@fer.unizg.hr

**Keywords:** symmetry-breaking, rigorous coupled wave analysis, glide symmetry, dielectric characterization

## Abstract

This paper introduces a novel method for measuring the dielectric permittivity of materials within the microwave and millimeter wave frequency ranges. The proposed approach, classified as a guided wave transmission system, employs a periodic transmission line structure characterized by mirror/glide symmetry. The dielectric permittivity is deduced by measuring the transmission properties of such structure when presence of the dielectric material breaks the inherent symmetry of the structure and consequently introduce a stopband in propagation characteristic. To explore the influence of symmetry breaking on propagation properties, an analytical dispersion equation, for both symmetries, is formulated using the Rigorous Coupled Wave Analysis (RCWA) combined with the matrix transverse resonance condition. Based on the analytical equation, an optimization procedure and linearized model for a sensing structure is obtained, specifically for X-band characterization of FR4 substrates. The theoretical results of the model are validated with full wave simulations and experimentally.

## 1. Introduction

With the advancement of microwave and millimeter wave technology, especially in applications such as telecommunications, radar systems, and satellite communications, the demand for high-quality components and devices has increased. In order to design complex structures and components, it is necessary to know the parameters of the materials from which they are made. The complex and often frequency-dependent nature of permittivity requires the use of specialized techniques to obtain accurate data over a wide range of conditions. The aim of this paper is to propose a new measurement method to determine the permittivity of the considered materials in the microwave and millimeter wave ranges.

The commonly used measurement methods can be divided into several categories: (a) probe reflection system, (b) cavity resonance system, (c) guided wave transmission system, (d) parallel plate system, and (e) free space method. Which method is most suitable for certain measurements depends on the properties of the sample, i.e., the size of the sample, whether it is solid or liquid, whether destructive measurements are permitted, how easy it is to prepare the sample, etc. Comparisons of the different methods can be found, e.g., in [1,2,3,4].

The symmetry properties of periodic structures have previously been used to determine the index of refraction values of dielectric layers in the optical frequency range. For example, if we put a metal film on a dielectric slab under test (SUT) a surface plasmon (SP) mode will be excited [5]. The propagation constant of the SP mode depends on the index of refraction of the SUT. If the sensor structure contains a periodic diffraction grating, then the intensity of the refracted field depends on whether the SP mode is excited, which can be used to determine the value of the index of refraction [6]. Additionally, if one makes a structure that has resonators with perturbed periodicity (e.g., asymmetrically positioned slits in silver resonators) Fano resonances are excited that depend on the refractive index of the material under sensing [7]. Using metamaterials, similar concepts can also be realized in the microwave frequency range [8].

The proposed measurement approach can be categorized as a guided wave transmission system; however, the transmission line under consideration is a periodic structure possessing glide symmetry. Glide symmetry is a representative of higher symmetries, as it is composed of two symmetry operations, translation and mirroring [9,10,11]. From a practical point of view, glide symmetry has the property of closing the first stopband in the dispersion diagram of the equivalent periodic structure with pure translational symmetry [12,13]. Thus, if the glide symmetry is broken by the presence of a measurement sample, the first stopband is opened again [14]. The cut-off frequencies of the formed stopband depend on the permittivity of the measurement sample, so that such a structure can be used to measure the permittivity of the material under consideration. Compared to conventional guided wave transmission systems, where the material sample is typically inserted into a waveguide [15,16], instead of measuring the transmission and reflection coefficients, we determine the stopband caused by the presence of the material sample. The proposed method offers a simpler setup for measuring dielectric permittivity compared to the resonant cavity-based methods, which are considered the most reliable [4]. Unlike the cavity based methods, which require specialty hardware (force gauges, steel clamp bars, cavity, etc.), the sensing unit for the proposed method can be manufactured using, a now widely available, 3D printing approach. Additionally, we believe it is well suited for scaling to higher frequencies, as the sensor’s length and width are preferred to be electrically large, and the thickness of the sensor can be changed by the choice of dielectric material and parameters of the grating structure.

## 2. Theoretical Background

**Figure 1:**
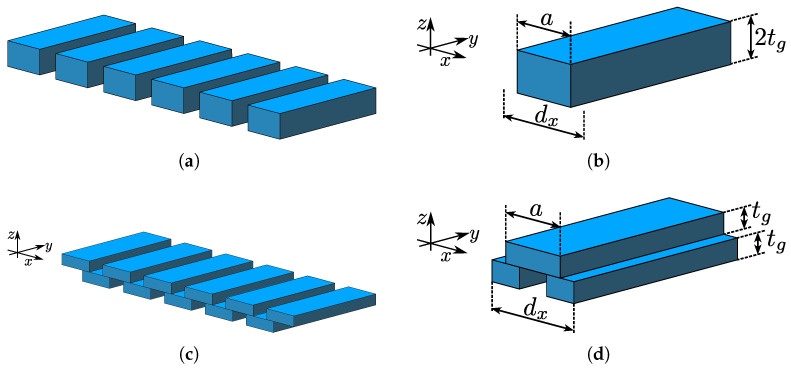
Segments of 1D binary dielectric gratings with (**a**) mirror and (**c**) glide symmetry truncated in the *y*-direction, but in the analysis they are considered infinite along this direction. The corresponding unit cells with physical dimension are shown in subfigures (**b**,**d**).

**Figure 2:**
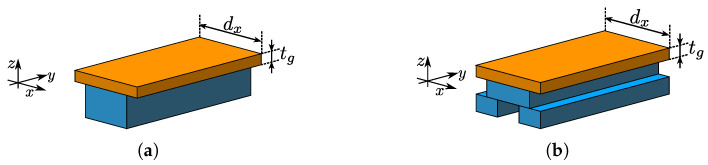
Unit cells of broken (**a**) mirror and (**b**) glide symmetric structures, shown in blue, due to presence of a dielectric slab, shown with orange color.

In this section, 1D periodic dielectric structures are modeled using Rigorous Coupled Wave Analysis (RCWA) [17,18,19,20]. The analysis will be limited to structures possessing either a mirror or glide symmetry, and the focus is to find the propagation properties, i.e., the dispersion diagram of such structures when their inherent symmetry is broken by the presence of a dielectric slab on one side. The hypothesis is that breaking the symmetry will change the propagation properties in such a way that it will be possible to evaluate the permittivity of the dielectric slab. Additionally, the change in propagation properties will be studied depending on which type of symmetry is broken. The examples of structures with mirror and glide symmetries that will be analyzed and the corresponding unit cells of broken symmetry cases are shown in Figure 1 and Figure 2, respectively.

Glide symmetric structures have been typically used for their properties inherited from the presence of higher symmetry and the fact that such symmetry closes the first stopband, making the structure less dispersive and more wideband [9,12,21]. It is expected that for small values of permittivity and for thin dielectric slabs, the change will be more pronounced in broken mirror (BM) structures than in broken glide (BG) structures. This is expected since there can be no abrupt change in propagation properties when breaking the glide symmetry with a small perturbation. In order to study these effects, an analytic dispersion equation is derived in this section for the breaking of different symmetries due to the presence of a dielectric slab. The equations are based on RCWA in combination with the matrix transverse resonance condition [20]:

(1) $$\det\;Z_{\mathrm{in}}^{\left(\mathrm{up}\right)}+Z_{\mathrm{in}}^{\left(\mathrm{dn}\right)}=0$$

where $Z_{\mathrm{in}}^{\left(\mathrm{up}\right)}$ and $Z_{\mathrm{in}}^{\left(\mathrm{dn}\right)}$ are input impedance matrices, on any arbitrary plane $z=z_{0}$, for waves propagating in ±z directions. The details of how to obtain the matrices $Z_{\mathrm{in}}^{\left(\mathrm{up}/\mathrm{dn}\right)}$ are given in the following subsection.

### 2.1. Dispersion Equations

RCWA is well suited for analyzing one-dimensional (1D) and two-dimensional (2D) periodic multilayer structures, under the constraint that the layers share the periodicity. For the analysis of 1D periodic structures along *x*-axis, transverse electric (TE) and transverse magnetic (TM) modes guided by the structure along the *x*-direction ($k_{y}=0$) can be examined separately. In the RCWA approach, the tangential components of the electric and magnetic fields within each layer are expressed as a series of spatial harmonics, each with a different complex amplitude, propagating perpendicular to the layers. This representation is obtained by solving the wave equation for a ${\mathrm{TE}}^{x}$ or ${\mathrm{TM}}^{x}$ polarized wave, by expanding it in the Bloch–Floquet basis. Using the Bloch–Floquet expansion, the wave equation for the TE case is

(2) $$\frac{d^{2}}{{\mathrm{dz}}^{2}}{\underline{E}}_{y}\left(z\right)=-k_{0}^{2}\left[{\underline{\underline{\eta }}}^{-1}-\frac{K_{x}^{2}}{k_{0}^{2}}\right]{\underline{E}}_{y}\left(z\right)$$

and for the TM case it is

(3) $$\frac{d^{2}}{{\mathrm{dz}}^{2}}{\underline{E}}_{x}\left(z\right)=-k_{0}^{2}\left[I-\frac{K_{x}\;{\underline{\underline{\epsilon }}}^{-1}\;K_{x}}{k_{0}^{2}}\right]{\underline{\underline{\eta }}}^{-1}{\underline{E}}_{x}\left(z\right)$$

where $K_{x}$ is a diagonal matrix containing the Bloch–Floquet wavenumbers $k_{x,m}=k_{x,0}+2\pi m/d_{x},\;m=-P,\ldots ,P$, with $k_{x,0}$ being the fundamental Bloch–Floquet wavenumber, and $d_{x}$ the periodicity of the structure. The number of Bloch–Floquet modes is truncated to (2P+1) and the choice of *P* depends on the structure under analysis, mainly depending on the contrast between the dielectric slabs. The elements of matrices denoted by $\underline{\underline{\eta }}$ and $\underline{\underline{\epsilon }}$ are given by

(4) $$\begin{array}{c} \eta_{mn}=\frac{1}{d_{x}}\int_{0}^{d_{x}}\frac{1}{\varepsilon (x)}e^{-j(k_{x,m}-k_{x,n})x}dx \end{array}$$

(5) $$\begin{array}{c} \epsilon_{mn}=\frac{1}{d_{x}}\int_{0}^{d_{x}}\varepsilon \left(x\right)e^{-j(k_{x,m}-k_{x,n})x}dx \end{array}$$

and are equal to the Fourier transform coefficients of the periodic permittivity functions ε(x) and 1/ε(x).

Equations (2) and (3) are Helmholtz matrix equations of the common form $d^{2}{\underline{E}}_{t}/{\mathrm{dz}}^{2}=-A{\underline{E}}_{t}$. Such a system of equations can be solved by eigendecomposition $A={\mathrm{QDQ}}^{-1}$. The subscript *t* stands as an abbreviation for the *tangential* and is replaced by *x* or *y* depending on the polarization of the wave with respect to the *x*-axis. The resulting eigenvalues are the elements of the diagonal matrix D and the physical interpretation of them is that they are equal to squared wavenumbers in the normal direction, that is, $\sqrt{d_{mm}}=k_{z,m}$. Solving Equations (2) and (3) in such a way allows us to write the tangential components of the electromagnetic (EM) field, grouped into vectors ${\underline{\phi }}_{t}\left(z\right)={\left[\phi_{t,-P},\cdots ,\phi_{t,P}\right]}^{T},\phi \in \{E,H\}$ with the entries corresponding to different Bloch–Floquet modes, as

(6) $$\begin{array}{c} {\underline{E}}_{t}\left(z\right)=Q\left[\exp\left(-jK_{z}z\right)\underline{f}+\exp\left(+jK_{z}z\right)\underline{g}\right] \end{array}$$

(7) $$\begin{array}{c} {\underline{H}}_{t}\left(z\right)=P\left[\exp\left(-jK_{z}z\right)\underline{f}-\exp\left(+jK_{z}z\right)\underline{g}\right]. \end{array}$$

with the vectors $\underline{f}$ and $\underline{g}$ representing the amplitudes of spatial harmonics along the direction normal to the periodic layer plane. The exact values of $\underline{f}$ and $\underline{g}$ are unknown and are determined by fulfilling the boundary conditions. The complex propagation constant of these vertical space harmonics is described by diagonal matrices $\exp(\pm jK_{z}z)$ whose *m*th diagonal element equals $\exp(\pm j\sqrt{d_{mm}}z)$. The matrix P is uniquely determined by Q and depends on the nature of the mode under analysis. It is given with

(8) $$P=\left\{\begin{array}{cc} {\left[I-\frac{K_{x}\;{\underline{\underline{\epsilon }}}^{-1}\;K_{x}}{k_{0}^{2}}\right]}^{-1}\frac{{\mathrm{QK}}_{z}}{\omega \mu_{0}} & ,\;\mathrm{for}\;\mathrm{TM}\;\mathrm{modes}\\ \frac{{\mathrm{QK}}_{z}}{\omega \mu_{0}} & ,\;\mathrm{for}\;\mathrm{TE}\;\mathrm{modes} \end{array}\right..$$

**Figure 3:**
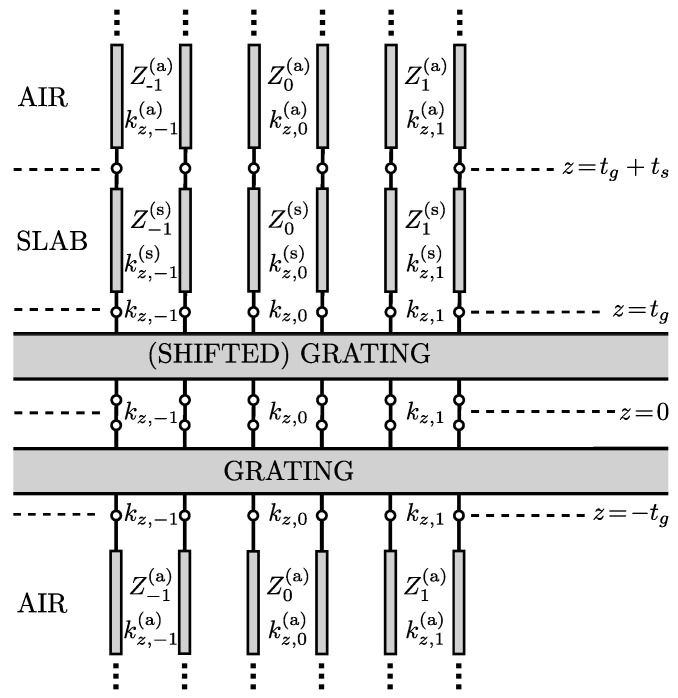
Equivalent transverse network representation of periodic structures with broken mirror/glide symmetry due to the presence of a dielectric slab, such as structures shown in Figure 2.

As demonstrated in [22], the tangential components of the fields in the glide-shifted layer can be expressed with expressions analogous to (6) and (7). The only modification is the substitution of Q, P, and $K_{z}$ with

(9) $$\begin{array}{c} Q^{\left(\mathrm{glide}\right)}=G⊙Q,\;P^{\left(\mathrm{glide}\right)}=G⊙P,\;K_{z}^{\left(\mathrm{glide}\right)}=K_{z} \end{array}$$

where matrix G is a symmetric square matrix whose elements on the main diagonal and all even diagonals are 1, while all odd diagonal elements are −1. The symbol ⊙ stands for the Hadamard product operator [23]. Now, the tangential components of the fields in nominal and glide-shifted layers can be obtained by solving only one Helmholtz equation. From expressions (6) and (7) and their glide analogues obtained with (9), together with the boundary conditions, it is possible to derive the input impedance matrices $Z_{\mathrm{in}}^{\left(\mathrm{up}/\mathrm{dn}\right)}$ for the transverse resonance condition. These will be obtained using the procedure given in [24] and representing the structures with equivalent multiport transverse networks. Figure 3 shows the generalized transverse resonance network for the BM and BG structures. The transverse multiport network primarily consists of two infinite-port components, which represent the two dielectric layers (in the BM case these two layers are identical). In this configuration, each port corresponds to a vertical space harmonic propagating along the *z*-direction with a wave number $k_{z,m}$, which are the diagonal elements of the $K_{z}$ matrix. To these ports are connected transmission lines with the slab region represented as a finite transmission line, and air half-spaces are represented with infinite transmission lines. Since the tangential components of the wavevector are identical in every layer, the vertical components $k_{z,m}^{\left(a/s\right)}$ depend on the layer. They are obtained from the invariance of the total length of the wavevector. Tangential components in the problem of propagation along the *x*-direction, which is being analyzed, are determined by the fundamental Bloch–Floquet wavenumber $k_{x,0}$, which leads to the expression

(10) $$k_{z,m}^{\left(a/s\right)}=\pm \sqrt{\varepsilon_{r}^{\left(a/s\right)}k_{0}^{2}-k_{x,m}^{2}}$$

for the vertical components of the wavevector in air and slab regions. Here, $\varepsilon_{r}$ depends on the material, thus it is equal to 1 if it is air, otherwise it is equal to $\varepsilon_{s}$. The choice of a sign for the square root of the complex number has to be made to ensure the attenuation wave at ±∞. The corresponding characteristic impedances in air or slab $Z_{m}^{\left(a/s\right)}$, written in matrix form, are

(11) $${\left(Z^{\left(a/s\right)}\right)}_{mn}=Z_{m}^{\left(a/s\right)}\delta_{mn}=\left\{\begin{array}{cc} \frac{\omega \mu_{0}}{k_{z,m}^{\left(a/s\right)}}\delta_{mn}\; & ,\;\mathrm{for}\;\mathrm{TE}\;\mathrm{modes}\\ \frac{k_{z,m}^{\left(a/s\right)}}{\omega \varepsilon_{0}\varepsilon_{r}^{\left(a/s\right)}}\delta_{mn}\; & ,\;\mathrm{for}\;\mathrm{TM}\;\mathrm{modes} \end{array}\right.\;$$

where $\delta_{mn}$ is the Kronecker delta symbol.

Input impedance matrices for up and down directions can be obtained using transmission line theory, and the full derivation is given in Appendix A. The final transverse resonance condition for both BM and BG structures can be simplified using the properties of Hadamard products with a matrix G proven in [22]. Since the derivation is long and cumbersome [25], it is omitted and only the resulting expression is given:

(12) $$\det\left[G^{\prime }\;⊙\;\left(Q\left[X^{\left(\mathrm{up}\right)}+X^{\left(\mathrm{dn}\right)}\right]P^{-1}\right)+G^{\prime \prime }\;⊙\;\left(Q\left[X^{\left(\mathrm{up}\right)}\pm X^{\left(\mathrm{dn}\right)}\right]P^{-1}\right)\right]=0$$

where the ± choice is based on symmetry, the − sign corresponds to BG, and the + sign to BM. Expression (A1) gives the definition of matrices $X^{\left(\mathrm{up}/\mathrm{dn}\right)}$. The matrices $G^{\prime }$ and $G^{\prime \prime }$ are constant binary matrices. The matrix $G^{\prime }$ contains ones on all odd diagonals and zeros on all even diagonals, while $G^{\prime \prime }$ exhibits the complementary pattern. Notice that the BM case is expanded to fit in a form similar to the BG case, but a simplified version of the transverse resonance condition for the BM case is

(13) $$\det\left[Q\left[X^{\left(\mathrm{up}\right)}+X^{\left(\mathrm{dn}\right)}\right]P^{-1}\right]=0.$$

The expanded form (12) better illustrates that the symmetry only affects odd indexed elements, that is, only combinations of Bloch–Floquet modes with indexes of different parity. This result suggests that for certain slab thicknesses $t_{s}$ and slab permittivities $\varepsilon_{s}$ the behaviors of complex solutions can be different.

### 2.2. Stopband Properties of Broken Symmetric Structures

To obtain the guiding properties of the considered sensing structure, the roots of an equation of the form $f\left(k_{x,0},k_{0}\right)=\det\left[M\left(k_{x,0},k_{0}\right)\right]=0$ where f:C×R→C and $M\in M_{\left(2P+1\right)}\left(C\right)$ must be found. Notice that *M* is given with Equation (12). For a given frequency $k_{0}$, we search for the fundamental Bloch–Floquet wavenumber $k_{x,0}=\beta_{x}-j\alpha_{x}$ that satisfies the dispersion Equation (12), thus obtaining the dispersion diagram $k_{0}\left(k_{x,0}\right)$ of the exact structure under analysis. The parts of the dispersion diagrams that are studied in this section are the first stopbands of TM modes, i.e., solutions of (3) that have $\alpha_{x}/k_{0}\neq 0$.

**Figure 4:**
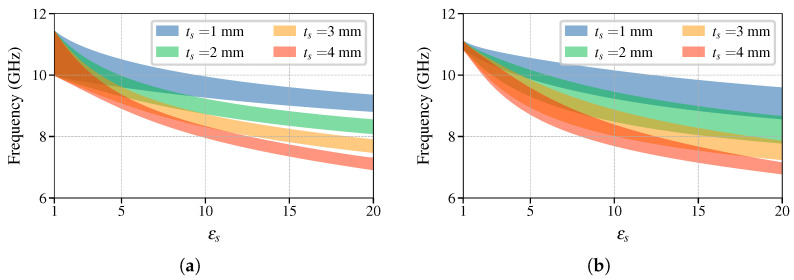
Parametric sweep of stopband width over the SUT permittivity $\varepsilon_{s}$ of (**a**) mirror- and (**b**) glide-symmetric binary grating. The shaded area corresponds to the first stopband frequency range.

To compare the stopband properties of the BM and BG structures, we consider binary dielectric gratings, illustrated in Figure 1, with the following parameters: $\varepsilon_{r}=10$, $d_{x}=8\;\mathrm{mm}$, a=7mm, and $t_{g}=3\;\mathrm{mm}$. The symmetry of the grating will be broken using a dielectric slab of varying thicknesses and permittivities. Variation in these parameters changes the frequency range of the stopband. Figure 4a,b show how the stopband frequency range changes with the change in dielectric permittivity of the SUT, $\varepsilon_{s}$, for the BM and BG symmetries, respectively. The stopband frequency range is marked as a shaded region. In both cases, for all the values studied of $t_{s}$ the frequencies are inversely proportional to $\varepsilon_{s}$. The difference is the width of the stopband and the curvature against $\varepsilon_{s}$ ($d^{2}/d\varepsilon_{s}^{2}$), both of which are more pronounced in the BG case.

**Figure 5:**
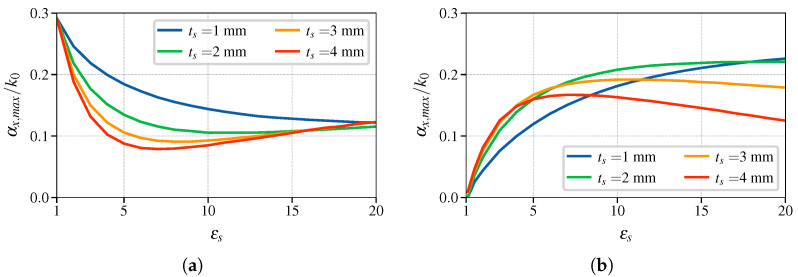
Parametric sweep of maximum value of attenuation constant $\alpha_{x,max}/k_{0}$ over the SUT permittivity $\varepsilon_{s}$ of (**a**) mirror- and (**b**) glide-symmetric binary grating.

The second parameter of the stopband that is studied is the maximum value of the attenuation constant $\alpha_{x,max}$. The significance of this parameter, in particular for sensor design, will be discussed in the following section. The dependence of $\alpha_{x,max}$ on $\varepsilon_{s}$ for the structure of BM and BG is shown in Figure 5a and Figure 5b, respectively. Breaking of the mirror symmetry reduces the $\alpha_{x,max}$ of the first stopband for all the cases, and they converge to approximately the same value. This suggests that the field is confined to the SUT and the grating is not significantly affecting the propagation. The initial slope is proportional to the thickness of the SUT, but for thicker substrates there is an inflection point. This confirms the hypothesis; the maximum attenuation in this case starts from 0 and increases as $\varepsilon_{s}$ increases.

This analysis was carried out under the assumption that the material under test does not have magnetic properties, that is, $\mu_{s}=1$. Similar analysis can be conducted for TE modes and the breaking of the symmetry affects the stopband properties in an analogous manner. The two modal solutions are independent; that is, their stopband properties are independent, but for both modes they are affected by permittivity $\varepsilon_{s}$ and by permeability $\mu_{s}$. To uniquely determine these parameters, two independent measurements are required. In the analytical model, these parameters appear only in $Z^{\left(\mathrm{SUT}\right)}$, specifically they appear in the characteristic impedance of transmission lines that model the slab under test and in the wavenumber describing the propagation within such a slab. In the wavenumber within the slab, they are multiplied together $k_{z,m}^{\left(s\right)}=\pm \sqrt{\mu_{s}\varepsilon_{s}k_{0}^{2}-k_{x,m}^{2}}$, that is, it is hard to distinguish the ratio of the two. However, they appear independent of each other in the characteristic impedances for the two modes, as given in (11). Now, it follows that, measuring the stopband starting frequency in the TE case in the same manner as described in the manuscript for the TM case (using the substrate with known permeability and known permittivity), a system of two independent equations with two unknowns can be constructed, and such a system can be solved to obtain $\varepsilon_{s}$ and $\mu_{s}$. The approximate model derived from RCWA would have to be extended to include the dependence on $\mu_{s}$, as well.

## 3. Sensor Design

The dependence of the stopband frequency range on the dielectric permittivity of the slab suggests that such a structure can be used for sensing purposes. One could take a finite section of the periodic structure, either mirror or glide symmetric, and place a dielectric slab of known thickness on top of it. Because the symmetry is broken, it is possible to uniquely determine the dielectric permittivity of the SUT. To determine the value of $\varepsilon_{s}$, it suffices to know only the frequency at which the stopband starts or ends, i.e., the cut-off frequency.

**Figure 6:**
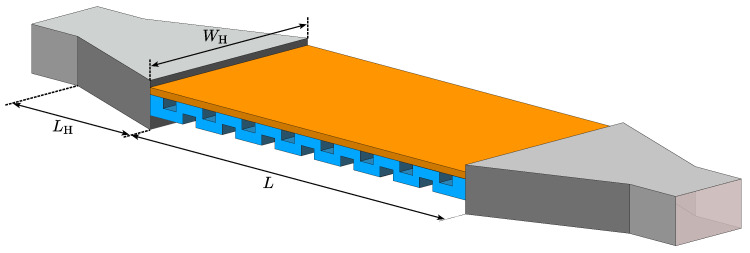
The illustration of the sensing setup with a broken glide sensor.

The sensing setup is made up of two identical H-plane sectoral horn antennas that are used to excite a TM wave within the periodic structure, as illustrated in Figure 6. The ports of the horn antennas are connected to the vector network analyzer, which is used to measure the scattering parameters $S_{ij}$. Since it is only necessary to measure the lowest/highest frequency of the stopband, it was empirically observed that the lowest frequency is least prone to deterioration from the finiteness of the structure and that it is also easier to determine it from measurements. The frequency of operation is dependent on the specific need, and the X-band frequency range is chosen as an example without loss of generality. The horn antennas used for excitation have dimensions $W_{H}=57$ mm and $L_{H}=46$ mm and are excited by a standard WR90 waveguide. The slab permittivity sweep in Figure 4 and Figure 5 spans a wide range of values, making it difficult to obtain an approximate expression relating the stopband start frequency $f_{SB,\mathrm{start}}$ and permittivity $\varepsilon_{s}$ for a given thickness $t_{s}$. If a limited range of permittivity values $\varepsilon_{s}$ is expected, then an approximation with a linear function can be good enough. Additionally, for a limited range of SUT parameters, it is possible to optimize the dielectric grating to achieve maximum resolution, that is, the change in $f_{SB,\mathrm{start}}$ is maximal.

Since the intention is to measure $S_{ij}$ parameters, in order to have easily distinguishable stopband properties such as strong reflection and low transmission with clear cut-off frequency, a high attenuation constant is desirable. The choice of symmetry is crucial for an efficient sensor design for a specific range of permittivity $\varepsilon_{s}$. The high attenuation constant additionally minimizes the necessary length of the sensor as well, which means that less material is required to manufacture it. The glide-symmetric case could be more convenient since it is easier to produce it in one piece, as depicted in Figure 6.

### 3.1. Optimization Procedure

As an illustration of the capabilities of the proposed measurements system, we would like to characterize the FR4 substrate. The dielectric permittivity of the FR4 substrates can vary from 3.8 to 4.8 depending on various parameters. Having an analytical expression for the dispersion Equation (12) makes it possible to implement an optimization procedure that would yield dimensions of the grating with the maximum change in $f_{SB,\mathrm{start}}$. The cost function *J* that is minimized in this procedure is as follows:

(14) $$J\left(a,d_{x},t_{g}\right)=\frac{1}{|\Delta f_{SB,\mathrm{start}}|}$$

where $\Delta f_{SB,\mathrm{start}}$ is the difference between $f_{SB,\mathrm{start}}$ for $\varepsilon_{s}=3.8$ and $\varepsilon_{s}=4.8$. In the optimization procedure, the dielectric permittivity of the periodic structure is fixed to $\varepsilon_{r}=10$, because the filaments available for 3D printing have predetermined values. Furthermore, optimization was limited to $t_{s}=1.5$ mm, which is a common thickness of the FR4 substrates. The dimensions obtained for the glide grating by this procedure are $d_{x}=8.7$ mm, a=6.3 mm, and $t_{g}=2.5$ mm, and the glide symmetric structure achieved higher resolution. If one wishes to extend the optimization to multiple values of $t_{s}$, the cost function could be modified to

(15) $$J\left(a,d_{x},t_{g}\right)=\sum_{i}\frac{w_{i}}{|\Delta f_{SB,\mathrm{start}}\left(t_{s,i}\right)|}$$

where $w_{i}$ are the weight coefficients for different substrate thicknesses.

**Figure 7:**
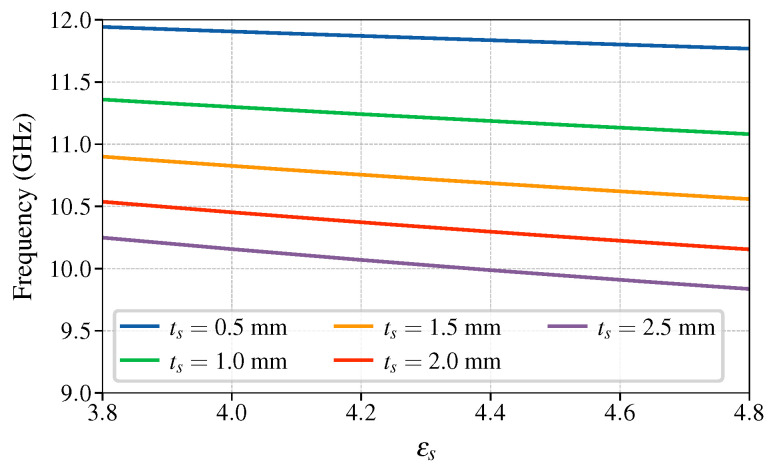
Stopband start frequency $f_{SB,\mathrm{start}}$ dependence on the SUT permittivity $\varepsilon_{s}$ for the optimized sensor in range of typical FR4 permittivities.

**Figure 8:**
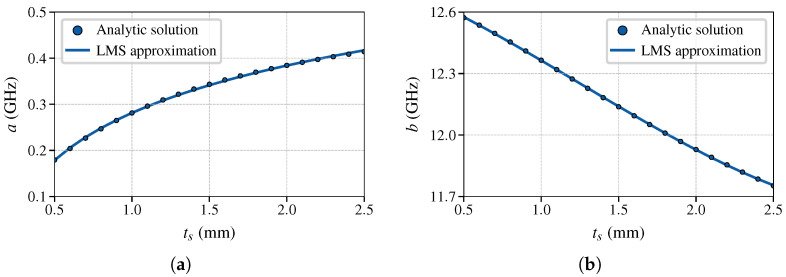
Dependence of coefficients (**a**) *a* and (**b**) *b* on thickness of SUT $t_{s}$ in the linearized expression for starting stopband frequency $f_{SB,\mathrm{start}}$. Markers denote analytically obtained results from the dispersion equation and solid line denotes the corresponding LMS approximations (17) and (18).

Characteristic curves of the optimized periodic structure are shown in Figure 7. As expected, with such a limited range of dielectric permittivities, $f_{SB,\mathrm{start}}$ depends linearly on $\varepsilon_{s}$ as $f_{SB,\mathrm{start}}\approx a\varepsilon_{s}+b$. The figure also indicates that the slope varies with the thickness, thus the resolution of the sensor deteriorates for thinner substrates. The range of thicknesses chosen is a typical range for an FR4 substrate; although FR4 comes in predetermined thicknesses, this continuous study gives more insight. The dependence of the slope *a* on the thickness is shown in Figure 8a where the markers show the slope of a straight line fitted through curves such as those in Figure 7. The frequency offset, the constant *b* in the linear equation, is shown in Figure 8b. Both *a* and *b* are approximated as a function of $t_{s}$ using the least mean squares (LMS) procedure and they are found to fit well to a logarithmic curve and a polynomial of third order, respectively, as

(16) $$\begin{array}{c} f_{SB,\mathrm{start}}\left(\varepsilon_{s},t_{s}\right)\approx a\left(t_{s}\right)\varepsilon_{s}+b\left(t_{s}\right) \end{array}$$

(17) $$\begin{array}{c} a\left(t_{s}\right)=\left(-0.147\ln\left(t_{s}/1\;\mathrm{mm}\right)-0.282\right)\;\mathrm{GHz} \end{array}$$

(18) $$\begin{array}{c} b\left(t_{s}\right)=\left(0.034t_{s}^{3}-0.131t_{s}^{2}-0.282t_{s}+12.742\right)\;\mathrm{GHz}. \end{array}$$

The polynomial of third order is required to achieve a good approximation for the given range of $\varepsilon_{s}$, for smaller ranges linear approximation can work just as well.

### 3.2. Numerical Validation

**Figure 9:**
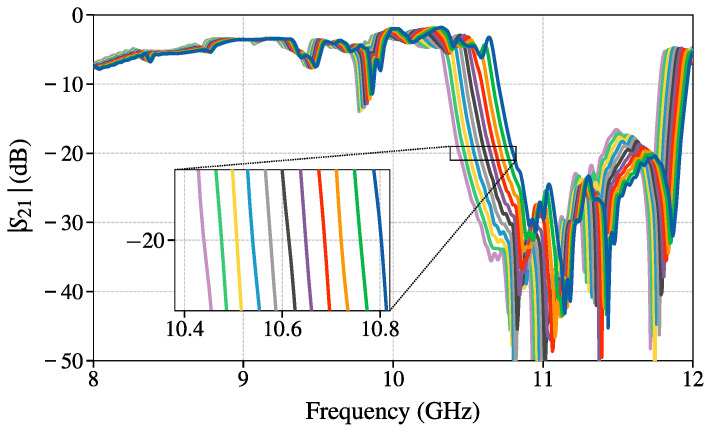
Full wave simulation results of sensing setup shown in Figure 6 for SUT permittivities ranging from 3.8 (blue) to 4.8 (purple) with step of 0.1.

To validate the concept illustrated in Figure 6, a full wave simulation of a finite structure is conducted in CST Studio Suite. The total length of the structure is 113 mm, which is 12 unit cells with small additional pieces of homogeneous dielectric material at both ends of the periodic structure. The SUT with thickness 1.5 mm is simulated with varying permittivity $\varepsilon_{s}$ from 3.8 to 4.8 with increments of 0.1. The resulting magnitude of the parameter $S_{21}$, shown in Figure 9, qualitatively agrees with the expected results shown with the orange line in Figure 7. The beginning of the stopband for all cases is in the frequency range between 10.5 GHz and 10.9 GHz. The criteria $S_{21}=const.$ for determining $f_{SB,\mathrm{start}}$ is not uniquely defined. This can be decided with one calibration measurement, where a dielectric slab of known permittivity would be used. All slopes have the same trend, so different choices of $S_{21}=const.$ criteria just shift the measured frequency and the spacing is preserved. This means that for example, the frequency point $S_{21}=-20$ dB can be measured for a slab of known permittivity $\varepsilon_{s,0}$, and just considering $\Delta f_{S21=-20}$ for SUTs with the same $t_{s}$, the parameter of interest $\varepsilon_{s}$ can be reliably obtained from (16) simply by measuring the $S_{21}=-20$ dB frequency for SUT. The only important parameter is the slope *a*, and the frequency offsets cancel each other. This effect is expected because the parameters *a* and *b* in (16) are functions of $t_{s}$ and the shift in frequency then is

(19) $$|f_{S21}-f_{S21,0}|\approx |a\left(t_{s}\right)\left(\varepsilon_{s}-\varepsilon_{s,0}\right)|$$

where $f_{S21,0}$ is the measured frequency in the calibration measurement. The inset of Figure 9 shows the segment zoomed in around $S_{21}=-20$ dB where any of the curves can be used as a calibration measurement. For the given model, the perfect match to the $f_{SB,\mathrm{start}}$ curve is obtained with criteria, $S_{21}=-22$ dB, but since this exact crossing point is not important criteria $S_{21}=-20$ dB will be used and the corresponding frequencies will be labeled $f_{S21}$. Furthermore, the same calibration measurement can be reused even for SUTs with different thicknesses, but Δb has to be introduced as an additional parameter that would account for the frequency change due to the selection of criteria $S_{21}$. The frequencies $f_{S21}$ for the curves in Figure 9 are given in Table 1. The approximation (19) is derived to be valid in the range of permittivities 3.8 to 4.8, and to test this, the average frequency shift is calculated for the neighboring crossings from Table 1. It is important to note that in this analysis we are taking $\varepsilon_{s}-\varepsilon_{s,0}=0.1$, i.e., we are analyzing the sensor structure in the opposite direction than the sensor would be used. To use the sensor, the change in frequency would be measured to estimate the change in permittivity. The obtained average shift in frequency, across the range of permittivities of interest with SUT thickness $t_{s}=1.5$ mm, for the change in permittivity of 0.1 is

(20) $$\Delta f_{S21}=\left(0.0363\pm 0.0044\right)\;\mathrm{GHz}.$$

This result agrees well with the approximation (19) that predicts 0.0342GHz shift across the whole permittivity range.

**Figure 10:**
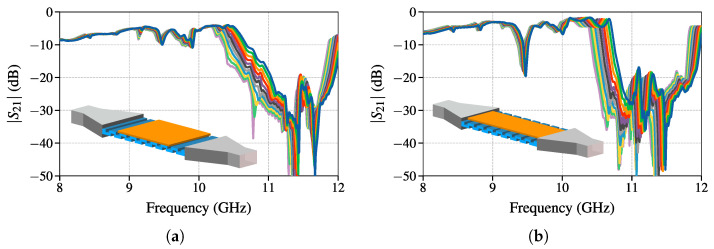
Full wave simulation results of sensing setup SUT permittivities ranging from 3.8 (blue) to 4.8 (purple) with step of 0.1 for (**a**) shorter SUT and (**b**) narrower SUT.

One might ask what is the minimum sample size of the SUT that can be measured. Figure 6 suggests that the width *W* and length *L* of the sensor should be the width $W_{\mathrm{SUT}}$ and the length $W_{\mathrm{SUT}}$ of the substrate under test. To consider these cases, additional full wave simulations are conducted. The first simulation is with $L_{\mathrm{SUT}}=80\;\mathrm{mm}\approx 0.7L$ referred to as “shorter” in subscript of the results, and the second is with $W_{\mathrm{SUT}}=40\;\mathrm{mm}\approx 0.66W$ referred to as “narrower” in the subscripts. The obtained $S_{21}$ parameters are shown in Figure 10a,b with insets illustrating how the truncated segments are aligned. The transmission properties of the shorter case have a lower slope when entering the stopband region, which is the result of a shorter segment that attenuates the wave. For this lower attenuation, it was necessary to take $S_{21}=-10$ dB as criteria for $f_{SB,\mathrm{start}}$ determination. For the narrower SUT sample case, the slope upon entering the stopband is preserved but with a slight frequency shift. In this case, no significant effect on the stopband region is the result of the majority of the energy being concentrated in the central part of the sensor. The narrower case exhibits a resonance just below the stopband, which is not to be confused with a stopband. The average changes in frequency $\Delta f_{S21}$ for an increase in $\varepsilon_{s}$ by 0.1 for these two cases are

(21) $$\begin{array}{c} \Delta f_{S21,\mathrm{short}}=\left(0.0335\pm 0.0062\right)\;\mathrm{GHz} \end{array}$$

(22) $$\begin{array}{c} \Delta f_{S21,\mathrm{narrow}}=\left(0.0328\pm 0.0024\right)\;\mathrm{GHz}. \end{array}$$

Both results are rather close to the results predicted by the theoretical model based on RCWA given with (19), but in the case of the shorter SUT the standard deviation is increased significantly. The reduced standard deviation in the narrower case is the result of $S_{21,\mathrm{narrow}}$ having a steeper slope when entering the stopband. And even though the stopband is slightly shifted, the model only considers the offset from the calibration frequency measurement. It is important to note that the calibration measurement has to be performed with a slab of the same width and length as the SUT.

In a real-world scenario, the frequency change is measured and from this measurement an estimate of the permittivity of the SUT $\varepsilon_{s}$ is made. The calibration substrate used can have a permittivity value outside the range considered for the linearized model, which is 3.8 to 4.8 in this case. The linearized model is essentially the first term of the Taylor series around $\varepsilon_{r}=4.3$, but using the RCWA it is possible to find the frequency shift from any permittivity $\varepsilon_{s,0}$ to 4.3 and just include it as a constant term in the final expression for $\Delta f_{S21}$. From this conclusion, by inverting (19), follows the expression for $\varepsilon_{r}$

(23) $$\varepsilon_{r}=\frac{4.3a+f_{S21}-f_{S21,0}-\Delta_{\mathrm{calibration}}}{a}=4.3-\frac{\Delta }{a}$$

where $\Delta_{\mathrm{calibraion}}$ is the change in stopband starting frequency $f_{SB,start}$ obtained by the RCWA when changing the permittivity from 4.3 to the permittivity of the calibration substrate. The final equation in (23) shows that the sensitivity of the proposed sensing structure, as defined in [5], depends only the thickness of the SUT, $t_{s}$, since the sensitivity is

(24) $$S=\frac{\delta (\Delta f_{S21})}{\delta \varepsilon_{s}}=a$$

and *a* is function only of $t_{s}$ given with (17). This means that the sensor is more sensitive when measuring thicker substrates.

### 3.3. Experimental Results

**Figure 11:**
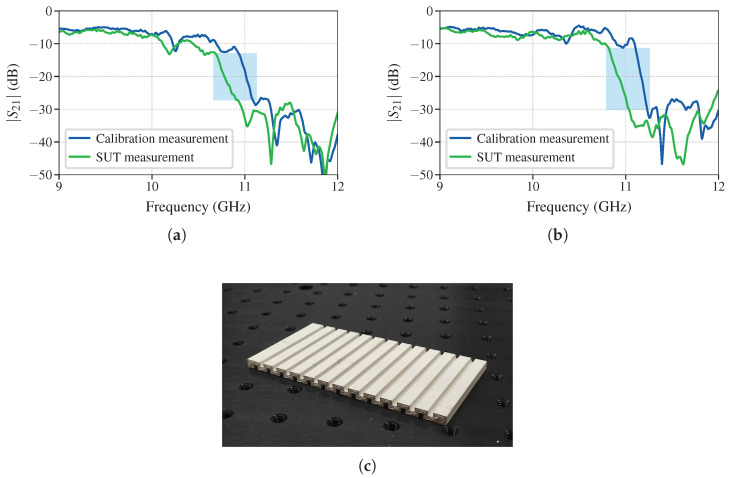
Transmission measurements of the (**a**) nominal and (**b**) narrow substrate. (**c**) The picture of the manufactured sensor.

Lastly, a prototype of the sensor is manufactured using Preperm ABS1000 dielectric filament [26] for a 3D printer with relative dielectric permittivity $\varepsilon_{r}=10$. The dimensions of the manufactured sensor are identical to dimensions of the one studied in Section 3.2, that is, L=113 mm, W=57 mm, $d_{x}=8.7$ mm, a=6.3 mm, and $t_{g}=2.5$ mm. This prototype is then used to characterize a sample of FR4 substrate. For the FR4 substrate, the relative dielectric permittivity of 4.26 is measured at 400 MHz using resonant approach. Two measurements are performed, one for the SUT with the same dimensions as the sensor $L_{\mathrm{SUT}}=57$ mm and one for a narrower SUT with $L_{\mathrm{SUT}}=48$ mm. For a calibration substrate, Taconic RF-35 is used, which has known relative permittivity of 3.5 at 10 GHz. In order to use (23), $\Delta_{\mathrm{calibration}}$ is calculated with RCWA to be 301.2 MHz. The obtained measurements for these two substrates and a picture of the prototype are shown in Figure 11.

From Figure 11a,b, it is clear that the slopes are not completely parallel, therefore the average value of the frequency shift for different values of $S_{21}$ is taken. The different slopes observed in the measurements can be attributed to the presence of losses in the material under test (FR4 is known to be quite lossy in the microwave frequency band). In periodic structures, the losses are not exhibited within the stopband, but they are most obvious at the beginning/end of the stopband. The range of $S_{21}$ values is taken to be the straight part of the slopes marking the beginning of the stopband. These ranges considered are highlighted with shaded areas in Figure 11a,b (approximately 150 points were used in the averaging process, depending on the measurement). From this averaged frequency shift, these two measurements, using (23), yield

(25) $$\begin{array}{cc} \varepsilon_{s}=\left(4.122\pm 0.025\right), & \;R=0.61\% \end{array}$$

(26) $$\begin{array}{cc} \varepsilon_{s,\mathrm{narrow}}=\left(4.136\pm 0.081\right), & \;R=1.95\%. \end{array}$$

These results have a slight discrepancy from the value of 4.264 measured at 400 MHz, but this decrease is in agreement with [27,28]. For FR4 substrates, it is observed that around 1 GHz the dispersion becomes quite pronounced. The authors in [27,28] report a decrease in relative permittivity of approximately 0.15 at 10 GHz from the value in the 100–500 MHz range. The measured decrease in relative permittivity of 0.142 and 0.128 in this article is therefore consistent with the results previously reported in the literature.

## 4. Conclusions

In this article, symmetry breaking effects on wave propagation through a dielectric grating are studied for cases of mirror and glide symmetry. The effects predicted by the analytic dispersion equation are leveraged to design a periodic structure that can be used to characterize dielectric materials. The design procedure of specific periodic structure is formulated as an optimization problem for a certain range of parameters that are expected to be measured. As an illustrative example, a sensor is optimized for the characterization of FR4 substrates for X-band frequency range. The linearized model of the sensor allows us to determine the dielectric permittivity of unknown sample after performing a calibration measurement with a substrate of known permittivity. The dimensions of the SUT are not limited strictly to the dimensions of the sensor but, as is shown, can be somewhat smaller in terms of both width and length. The proposed methodology can be extended to any frequency band of interest, but the limitation may be the resolution of the manufacturing process.

## Figures and Tables

**Table 1:** Frequencies selected by the $S_{21}=const$ dB criteria of nominal, shorter, and narrower full wave simulations.

$\varepsilon_{s}$	$f_{S21}$ (GHz)	$f_{S21,\mathrm{short}}$ (GHz)	$f_{S21,\mathrm{narrow}}$ (GHz)	$\varepsilon_{s}$	$f_{S21}$ (GHz)	$f_{S21,\mathrm{short}}$ (GHz)	$f_{S21,\mathrm{narrow}}$ (GHz)
3.8	10.803	10.680	10.744	4.4	10.576	10.464	10.539
3.9	10.760	10.646	10.708	4.5	10.539	10.426	10.507
4.0	10.720	10.613	10.671	4.6	10.507	10.397	10.476
4.1	10.687	10.576	10.636	4.7	10.476	10.380	10.445
4.2	10.648	10.536	10.603	4.8	10.440	10.345	10.416
4.3	10.606	10.502	10.572				

## Data Availability

The data presented in this study is available from the corresponding author upon request.

## Abbreviations

The following abbreviations are used in this manuscript:

RCWA	Rigorous Coupled Wave Analysis
BM	Broken mirror
BG	Broken glide
TE	Transverse electric
TM	Transverse magnetic
SUT	Substrate under test
LMS	Least mean squares

## Appendix A

The transmission line theory is used in matrix form where different modes are placed on the diagonal elements of the impedance matrices. This can be achieved because the waves propagating in homogeneous medium are orthogonal, i.e., there is no coupling between them. The input impedances in the glide-shift plane, z=0, are

(A1) $$\begin{array}{c} Z_{\mathrm{in}}^{\left(\mathrm{up}/\mathrm{dn}\right)}=Q\left[I+{\underline{\underline{\Gamma }}}^{\left(\mathrm{up}/\mathrm{dn}\right)}\;\right]{\left[I-{\underline{\underline{\Gamma }}}^{\left(\mathrm{up}/\mathrm{dn}\right)}\;\right]}^{-1}P^{-1}=QX^{\left(\mathrm{up}/\mathrm{dn}\right)}P^{-1} \end{array}$$

(A2) $$\begin{array}{c} {\underline{\underline{\Gamma }}}^{\left(\mathrm{dn}\right)}=-\exp\left(-jK_{z}t_{g}\right){\left[Q+Z_{\mathrm{in}}^{\left(a\right)}P\right]}^{-1}\left[Q-Z_{\mathrm{in}}^{\left(a\right)}P\right]\exp\left(-jK_{z}t_{g}\right) \end{array}$$

(A3) $$\begin{array}{c} {\underline{\underline{\Gamma }}}^{\left(\mathrm{up}\right)}=-\exp\left(-jK_{z}t_{g}\right){\left[Q+Z_{\mathrm{in}}^{\left(\mathrm{SUT}\right)}P\right]}^{-1}\left[Q-Z_{\mathrm{in}}^{\left(\mathrm{SUT}\right)}P\right]\exp\left(-jK_{z}t_{g}\right) \end{array}$$

where matrices ${\underline{\underline{\Gamma }}}^{\left(\mathrm{up}/\mathrm{dn}\right)}$ are reflection matrices that describe the ratio of complex amplitudes of vertical space harmonics $\underline{f}$ and $\underline{g}$. They are obtained by enforcing the continuity of the tangential components of the electric field at the planes $z=t_{g}$ and $z=-t_{g}$. This is performed with matrices $Z_{\mathrm{in}}^{\left(a\right)}$ and $Z_{\mathrm{in}}^{\left(\mathrm{SUT}\right)}$, which are input impedance matrices when looking into air and into a dielectric slab backed by air (*slab under test—SUT*), respectively. The transmission lines for the lower air half-space are infinite, as shown in Figure 3, and elements of the input impedance matrix $Z_{\mathrm{in}}^{\left(a\right)}$ are equal to the characteristic impedance of the transmission line for the corresponding $k_{z,m}^{\left(a\right)}$ given with (11). The following is the input impedance matrix into the thin dielectric layer of the slab: $Z_{\mathrm{in}}^{\left(\mathrm{SUT}\right)}$

(A4) $$\begin{array}{c} Z_{\mathrm{in}}^{\left(\mathrm{SUT}\right)}=\left[I+{\underline{\underline{\Gamma }}}^{\left(\mathrm{SUT}\right)}\;\right]{\left[I-{\underline{\underline{\Gamma }}}^{\left(\mathrm{SUT}\right)}\;\right]}^{-1}Z^{\left(s\right)} \end{array}$$

(A5) $$\begin{array}{c} {\underline{\underline{\Gamma }}}^{\left(\mathrm{SUT}\right)}=-\exp\left(-jK_{z}^{\left(s\right)}t_{s}\right){\left[Z^{\left(s\right)}+Z^{\left(a\right)}\right]}^{-1}\left[Z^{\left(s\right)}-Z^{\left(a\right)}\right]\exp\left(-jK_{z}^{\left(s\right)}t_{s}\right) \end{array}$$

where now $\exp(-jK_{z}^{\left(s\right)}t_{s})$ represents a diagonal matrix with elements $\exp(-jk_{z,m}^{\left(s\right)}z)$ entries along the diagonal.
